# Annotations

**Published:** 1911-02-18

**Authors:** 


### Annotations.

### Monument to Van Beneden.

An influential committee has been formed with the object of collecting subscriptions to defray the cost of erecting a worthy monument to the memory of the illustrious Belgian morphologist, Van Beneden. M. Cerfontaine, of the Institute of Zoology, quai des Pêcheurs, Liège, Belgium, secretary to the fund, will be glad to receive subscriptions. A subscription of 10 francs will give the right to a copy of the *liber memorialis*, the portrait of the scientist, and a list of subscribers; one of 30 francs, to the book and a bronze medallion portrait of the deceased, by the sculptor, G. Devreese; and a still higher one, not yet definitely settled upon, to a silver medal of larger size, by the same sculptor, as well as the book, etc.

### The First Female Doctor of Athens.

It is interesting to note from an article which appears in the *Frauen Rundschau* that the practice of impersonating men is not the exclusive prerogative of nineteenth-century women. There existed a law at Athens forbidding the exercise of the art of healing by women. The story goes that one day a good-looking youth presented himself before the famous physician, Hierophytes, demanding to be enrolled among those to whom he taught the science of medicine. Since he seemed to possess every qualification demanded of those desirous of becoming students of that science—namely, legitimate birth, celibacy, and the masculine sex—the youth was accepted and very quickly earned the approbation of the old physician by his extraordinary zeal. It was noticed, however, after a time that he rigidly confined his ministrations to patients of the gentler sex, with whom he became so popular that his brother practitioners began to feel that his success was reflected on their pockets. Believing that his powers of fascination were to blame for this, they haled him before the magistrates on a charge of seducing his lady clients. The victim of their prosecution was perfectly cool when face to face with his judges, merely smiling and remarking that the charge was ridiculous, inasmuch as he was a woman! The prosecution broke down completely, but matters could not remain at a standstill, since, by exercising the art of medicine as a woman, the "youth" had let himself in for a death penalty, according to Athenian law. He was, however, not deserted by his clients, for the women of Athens formed a league to defend him, and ultimately obtained his release. Later the law itself was altered, and the first female physician of Athens allowed to resume her profession.

### A Functionary of Napoleon III.

The recollections of anyone, however remotely, connected with Royalty will always find a public, and, therefore, there is no need to apologise for recording the fact that the memoirs of Dr. John Evans, sometime dentist to Napoleon III., are expected to be published. This work will lose much by his death, which removes not only a capable dental surgeon, but one who figured in many well-known circles in his day. There was indeed more than one romantic fact about Dr. John Evans: the lifelong residence of an Englishman in Paris is always that; but his disused title of Marquis D'Oyley added a certain cachet to a man who possessed a peculiar individuality. He followed Napoleon III. to Italy, and saw active service again at Sedan, where he was busy among the ambulance. The Emperor is said to have decorated him with the Commandership of the Legion of Honour in one of his last investitures. The death of such a man removes a landmark; we are more cosmopolitan and less of court functionaries to-day, one more thread with a past generation is snapped. Lady Cardigan's memoirs gave the public a valuable flavour of that, being, in fact, an eighteenth-century book born out of due time; these of Dr. John Evans perhaps might supplement her record as another side dealing only with a slightly later period.

### The Encyclopædia Britannica.

The eleventh edition of "The Encyclopædia Britannica," which is being published by the Cambridge University Press, is now nearing completion, and promises to surpass its predecessors in every way. The individual writers who are responsible for the subjects most likely to interest readers of The Hospital—medicine and institutional science—are recognised experts in their several departments, and every care has been taken to make the edition thoroughly up to date. The statistics with regard to any new edition of such an enormous work as a really useful encyclopædia are always instructive, and no one can be astonished when he hears that the present edition has been completed at the cost of half a million pounds sterling. After that it is fairly easy to grasp the other figures—six miles of pages, twelve miles of piled books, equalling two Mount Everests superimposed on a Snowdon; 1,500 tons of India paper, needing a train of vans six miles long for its transport—and all this so ably arranged that the final turnout, the completed set bound in sheepskin, can be obtained for £30! As a literary investment an en-encyclopædia the "Britannica" has special claims titioner and the harried institutional worker: as an encyclopædia the "Britannica" has special claims on both. It is not only a great work in the sense that it is materially huge; it is equally stupendous as a monument of patient elaboration and as patient condensation which should appeal to everyone.

### A Notice.

With reference to a paragraph in the issue of The Hospital for December 17, 1910, entitled "An Advance Announcement," in which comments were made upon an advertisement of a new book by Mr. H. C. Ross, we desire to state that we are fully satisfied that Mr. Ross was in no way responsible for, or had knowledge of, the advertisement in question, and to withdraw unreservedly anything in the paragraph which might be calculated to give a contrary impression. We wish, at the same time, to express our great regret to Mr. Ross for any injury or annoyance that our comments may have caused him.

